# Integrated Continuous Wet Granulation and Drying: Process Evaluation and Comparison with Batch Processing

**DOI:** 10.3390/pharmaceutics15092317

**Published:** 2023-09-14

**Authors:** Seth P. Forster, Erin Dippold, Abbe Haser, Daniel Emanuele, Robin Meier

**Affiliations:** 1 Merck & Co., Inc., West Point, PA 19486, USA; 2 Organon & Co., Inc., Jersey City, NJ 07302, USA; 3 L.B. Bohle Maschinen und Verfahren GmbH, 59320 Ennigerloh, Germany; d.emanuele@lbbohle.de (D.E.); r.meier@lbbohle.de (R.M.)

**Keywords:** continuous granulation, wet granulation, continuous manufacturing, continuous drying, pharmaceutical, pharmaceutical manufacturing

## Abstract

The pharmaceutical industry is in the midst of a transition from traditional batch processes to continuous manufacturing. However, the challenges in making this transition vary depending on the selected manufacturing process. Compared with other oral solid dosage processes, wet granulation has been challenging to move towards continuous processing since traditional equipment has been predominantly strictly batch, instead of readily adapted to material flow such as dry granulation or tablet compression, and there have been few equipment options for continuous granule drying. Recently, pilot and commercial scale equipment combining a twin-screw wet granulator and a novel horizontal vibratory fluid-bed dryer have been developed. This study describes the process space of that equipment and compares the granules produced with batch high-shear and fluid-bed wet granulation processes. The results of this evaluation demonstrate that the equipment works across a range of formulations, effectively granulates and dries, and produces granules of similar or improved quality to batch wet granulation and drying.

## 1. Introduction

### 1.1. Continuous Manufacturing of Pharmaceutical Oral Drug Products

The pharmaceutical industry has started to invest in the commercial scale continuous manufacturing (CM) of drug substances and drug products driven by their potential for operational flexibility and improvements in product quality [1]. With support and advocacy from the U.S. FDA [2] and other regulatory authorities, including the adoption of the ICH Q13 guidance [3], more filings are incorporating continuous processing. A recent article from the FDA compared five oral solid dosage (OSD) products using CM filed from 2015–2022 against reference batch filings and found that CM filings reached the market 4–12 months sooner than batch products, with an estimated $171–537 M revenue benefit [4]. The FDA also found that the CM filings did not have equipment, process, or batch size changes in post-approval supplements, though there were many submissions with batch processes.

Novel equipment and process development has allowed for the wider application of CM to OSD products. For example, loss-in-weight feeders with improved control at reduced mass feed rates [5], novel tablet coating equipment designs [6], integrated granulation and tablet compression lines [7], and a wide variety of process analytical tools (PAT) enable CM in production. Progress in equipment and control systems is now leading to further studies to extend the duration of processing [8] and to develop more sophisticated and validated process control [9].

### 1.2. Wet Granulation Advantages and Development of Continuous Wet Granulation

Wet granulation (WG) is used to achieve a high drug loading, for modified release formulations, or to incorporate liquid excipients. WG formulations can reduce the risk of API segregation, improve flow or improve compactibility compared with direct compression or dry granulation. In one survey, WG was used for 38% of tablet and capsule formulations overall and 61% of formulations with fine API [10]. A large data set from one pharmaceutical company showed that WG was often used for poorly flowing APIs or to achieve higher drug loadings [11]. However, among conventional OSD processes, batch wet granulation requires more processing steps and larger equipment installations and GMP facilities. Typically, high-shear wet granulation (HSWG) with fluid-bed drying (FBD) or fluid-bed granulation (FBG) equipment is used, which requires much larger bowls and drying chambers upon scale-up. This complexity often creates challenges during the development and scale-up of both batch and continuous wet granulation.

Twin-screw wet granulation (TSWG) is a highly productive continuous means of wet granulation. The initial development of TSWG technology for pharmaceuticals used the same twin-screw extruders, which are used to produce pharmaceutical amorphous solid dispersions and before that to compound polymers and in the food industry [12]. Over time, the granulation equipment has been refined to better meet the requirements of wet granulation and research on the impact of material attributes and process parameters has enabled a better understanding of the TSWG process [13,14].

### 1.3. Continuous Drying vs. Continuous Granulation with Batch Drying

A key gap in the implementation of continuous wet granulation is the ability to effectively continuously dry granules. While the material can be dried within the extruder barrel [15], this requires somewhat higher processing temperatures and may not be a possible mechanism for API with temperature sensitivity. Until recently, continuous fluid-bed dryers tended to be large installations with a high amount of hold-up limiting application at the small scale; for example, long, horizontal, or multicell fluid beds used to dry food products [16]. More recently, semi-continuous, segmented fluid-bed systems have been implemented with pharmaceutical continuous granulation lines at the production scale [17], though the filter cleaning and scalability may be challenging [18].

**Figure 1:**
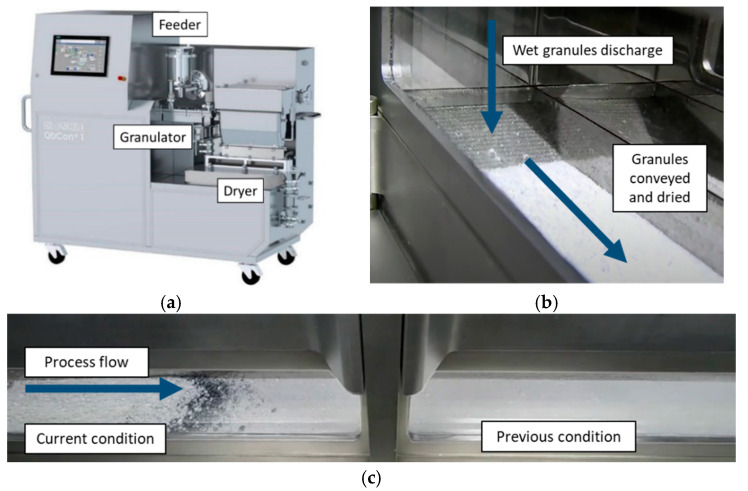
(**a**) QbCon 1 granulator and dryer equipment, (**b**) point of transfer to continuous dryer, (**c**) continuous dryer showing limited back-mixing between process conditions.

Recently, a novel, microfluidized vibratory fluid-bed dryer (VFBD) was designed with demonstrated efficient, continuous drying (QbCon^®^ 1, L.B. Bohle, Ennigerloh, Germany) [19]. This equipment, as shown in Figure 1, was designed for plug flow, which has little back-mixing and has easy visibility into the dryer to qualitatively assess granule quality and aid in troubleshooting. The wet granules are continuously discharged into the integrated vibratory fluid-bed dryer (VFBD). Dry compressed air is heated within the unit and metered into the dryer. The perforated bed vibrates at a controlled acceleration rate to gently fluidize the granules and convey them through the dryer. Filters within the dryer are regularly cleaned using compressed air. VFBD is much faster than batch drying, complete within several minutes, and more gently, with less granule agitation, than typical FBD. This process has also been successfully scaled to the commercial scale [20]. The goal of this work is to compare placebo and active granulations made by batch processes (HSWG/FBD or FBG) by continuous granulation with batch drying (TSWG to tray drying) and by continuous granulation and drying (QbCon 1). This technical process and product quality evaluation will inform process selection for future products.

## 2. Materials and Methods

APIs were manufactured by Merck. MCC (Avicel^®^ PH102, Rahway, NJ, USA) and croscarmellose sodium (Ac-Di-Sol^®^) were supplied by Dow/DuPont, now IFF, Midland, MI, USA. Lactose monohydrate 312 was supplied by Kerry, Norwich, NY, USA. PVP (Kollidon^®^ K25) was supplied by BASF, Ludwigshafen, Germany. Magnesium stearate (Hyqual^TM^) was manufactured by Mallinckrodt, St. Louis, MO, USA.

### 2.1. Formulations

In order to understand the impact of excipient and API properties, several formulations were evaluated in this study:

(1) A placebo formulation of conventional wet granulation excipients microcrystalline cellulose (MCC) and lactose (Table 1); (2) a formulation of a hydrophilic API, MK-A, with a high level of lactose monohydrate, known to readily granulate (Table 2); and (3) two formulations of a hydrophobic API, MK-B (Table 3 and Table 4). These formulations were studied because of prior experience with batch wet granulation at pilot and commercial scale for clinical development, prior twin-screw wet granulation evaluation with the Leistritz 18 mm, and the availability of bulk quantities of the APIs. A separate MK-B formulation (Table 4) with more MCC was also studied with the intention of improving the performance of the formulation over the reference formulation. MCC can absorb water, so it was expected that high-quality granules could be made at lower granulation fluid levels (GFL), also known as liquid-to-solid ratios (L/S). L/S is reported as the ratio of the total binder liquid feed rate to the dry solid feed rate.

### 2.2. Continuous Granulation and Drying

**Figure 2:**
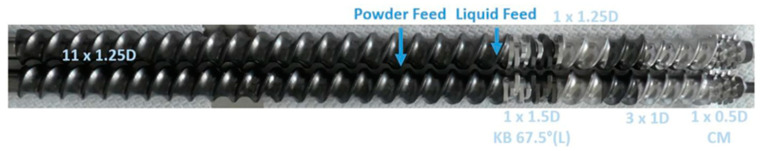
Screw profile used for TSWG on QbCon1. D = Diameter, indicating how the length of the screw element compares with the granulator screw diameter; KB = kneading block element; CM = combing element.

This work focuses on continuous granulation and drying (QbCon 1, L.B. Bohle, Ennigerloh, Germany) process performance, output granule properties, and downstream tabletability. The QbCon 1 granulation process used the screw and barrel profiles shown in Figure 2 below. Pre-blends were prepared by tumble blending in approximately 10 kg batches within a 40 L tote blender (LMA-40, L.B.Bohle, Ennigerloh, Germany) for 10 min at 26 rpm. The pre-blends were charged into an integrated loss-in-weight feeder (GZD150.12, Gericke, Regensdorf, Switzerland) feeding into the granulator barrel under gravimetric control at the set points indicated. Binder solutions were prepared at the proper concentration to achieve the desired PVP concentration at the target L/S level, from 11–29% *w*/*w*. The binder solution was metered into the barrel using an integrated pump. The liquid and solid feed rates and screw speed for granulation were adjusted to different conditions. The wet granules were discharged for several minutes until they appeared visually consistent and then they were fed into the continuous dryer. The dryer air temperature, air flow rate, and vibration acceleration were then adjusted so that the dried granules were less than 2% loss on drying (LOD). The vibration rate is controlled as a dryer acceleration setting, with adjustments based on a qualitative assessment of fluidization and bed height. The process setpoints and resulting LODs are listed in Table 5.

Residence time distribution (RTD) through the VFBD was measured by a tracer study using blue granules made using the 20% *w*/*w* MCC placebo formulation where blue dye was added to the feed of the granulator and collected. The blue granules were manually added to the dryer and the discharge color was measured using a camera and image analysis software (ExtruVis 3, MeltPrep GmbH, Graz, Austria). Residence time distributions (RTDs) in the dryer were measured across a typical range of acceleration settings, from 4–8 m/s^2^, with constant dryer airflow.

Dried granules were tested for LOD (HR73 Halogen moisture analyzer, Mettler-Toledo HR 73, Columbus, OH, USA) with the endpoint at weight change <1 mg over a 140 s run time, with a temperature of 90 °C. After the LOD was confirmed, dried granules were discharged to waste for approximately 5 min and then collected in bulk for downstream processing.

These results were compared to granules that were previously made by other granulation processes: (1) Continuous twin-screw wet granulation (Leistritz 18 mm, Somerville, NJ, USA) granulated at 0.9 kg/h with 5% L/S at 450 rpm for MK-A and a 2 kg/h solid feed rate with 15–25% L/S at 200 rpm for MK-B F2, followed by batch tray drying (Isotemp^®^ oven, Fisher Scientific, Dubuque, IA, USA); (2) Previously made batch granulated granules. The reference MK-A batch granulated formulation was granulated using FBG with a 450–550 cfm inlet air flow, a 240–270 g/min spray rate, a 60 °C inlet air temperature, and dried <1.5% LOD. This batch was made at a 70 kg scale using a Niro^®^ MP-4 (GEA, Columbia, MD, USA). The reference MK-B batch process formulation was granulated using HSWG at a 5 kg scale with wet binder addition to 17% L/S using a 25 L bowl (Diosna Dierks & Söhne GmbH, Osnabrück, Germany) followed by fluid-bed drying (Niro^®^ MP-1, GEA, Columbia, MD, USA) to <2% LOD using 60 °C inlet air with 425–550 cfm airflow. The processing conditions for batch granulation and drying were based on previously developed large-scale conditions for these products.

### 2.3. Product Performance Characterization

#### 2.3.1. Granule Size Distributions

The dried, unmilled granules were measured in triplicate using dynamic image analysis (QICPIC with RODOS, Sympatec, Inc., Pennington, NJ, USA) with vibratory dry feed (VIBRI), 2 bar dispersion pressure, and an M7 measuring range. The reported particle size attributes are based on volume mean distributions.

#### 2.3.2. Scanning Electron Microscopy (SEM)

Environmental scanning electron microscopy (SEM) was performed at low vacuum with a 20 kV accelerating voltage and 3.0 nm spot size using a Large-Field Detector on a Quanta FEG 250 (FEI, Hillsboro, OR, USA). The samples were not sputter coated.

#### 2.3.3. Bulk/Tap Densities

Bulk and tap density were measured using approximately 10 g of granules in a 25 mL graduated cylinder. Tapping was performed 500 times (Autotap, Anton Paar QuantaTec Inc., Boynton Beach, FL, USA). From these data, the Carr index (Equation (1)) was calculated to describe the flow behavior of the granules.

(1) $$Carr\;Index=100\times \frac{Tapped\;density-Bulk\;density}{Tapped\;density}$$

#### 2.3.4. Tabletability

Tableting blends of unmilled test granules were prepared in glass bottles using tumble blending (TURBULA T2F, Willy A. Bachofen AG, Muttenz, Switzerland) for 5 min at 46 rpm followed by lubrication with #30 sieved magnesium stearate for 3 min at 46 rpm.

Tablets were manufactured using a 3/8″-diameter, round, flat-faced punch. The tablet blend was compressed at different compaction forces using a compaction simulator (Roland Research Devices, Inc., Ewing, NJ, USA) and the resulting tablets were tested for weight, thickness, and hardness (Dr. Schleuniger, Sotax, Inc., Westborough, MA, USA). Formulation tabletability was estimated based on the tablet tensile strength (Equation (2)) versus compaction pressure [21].

(2) $$TS=\frac{2\times tablet\;hardness\;(N)}{\pi \times tablet\;diameter\;\left(mm\right)\times tablet\;thickness\;(mm)}$$

## 3. Results and Discussion

### 3.1. Granulation and Drying Process Performance

Granules were successfully produced across a wide range of process parameters. Qualitatively, lower liquid levels were able to achieve the same extent of granulation as observed with batch granulation. The granulator and dryer designs allow for the rapid adjustment of process parameters without cleaning between conditions. A condition can be tested within a few minutes instead of taking several hours to set up, run, and clean pilot-scale batch granulators or dryers. Benchmarking against internal batch processing indicated that evaluating these conditions would take more than 30 working days versus 4 for continuous processing.

The granules were readily dried to <2% LOD by adjusting the process airflow or temperature. Table 5 shows the dryer conditions used for these trials, showing the dryer air temperature and air flow increase required for drying. The granules are conveyed through the dryer by the vertical vibration of a dryer plate. The granules flow through the system in nearly plug flow, with little back-mixing. Process air and the plate vibration assist in granule fluidization.

#### Dryer Residence Time Distributions

The mean residence times decreased with increasing acceleration, roughly inversely to the square of acceleration (Table 6). The RTDs are narrow, with approximately 90% of the residence times within 20% of the mean residence time, indicating that plug flow of the granules can nearly be achieved across the studied range.

### 3.2. Granule Characterization

#### 3.2.1. Granule Size Distributions

Previous reports [22] have related the extent of granulation to (1) L/S, (2) the relative barrel fill level, estimated in this case by the specific throughput, ST = solid feed rate/screw speed, and (3) the specific mechanical energy, SME = 2π × torque × screw speed/mass flow rate.

**Figure 3:**
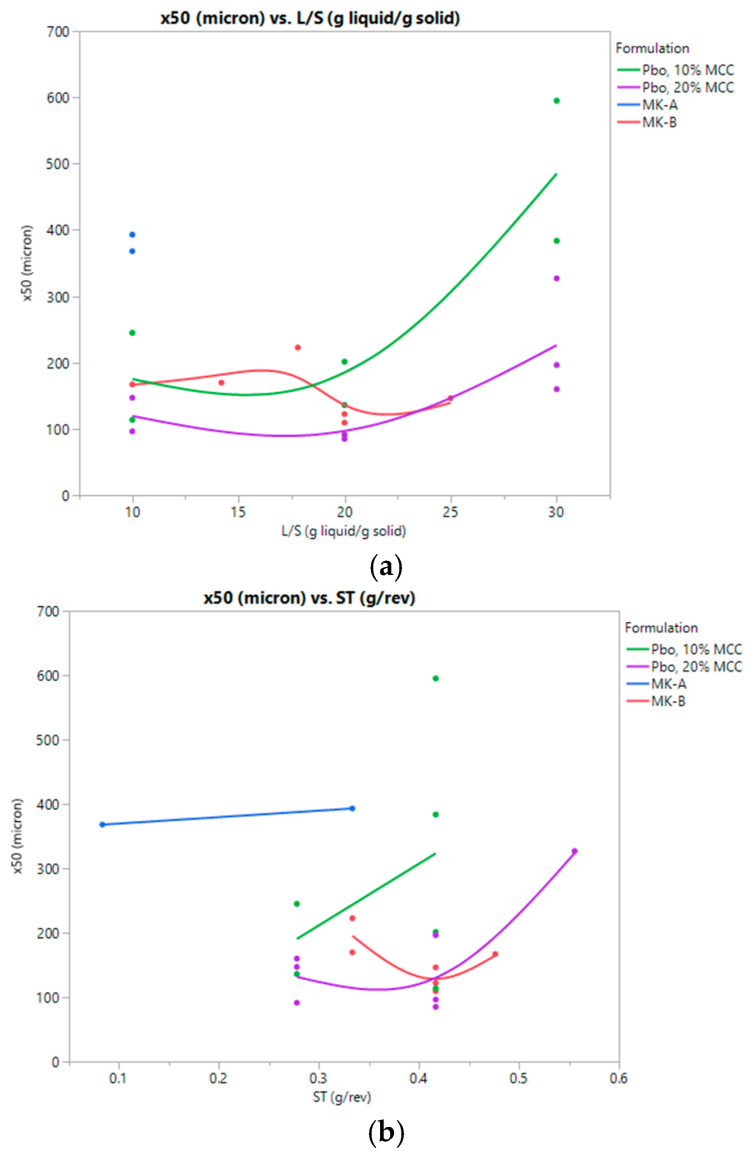

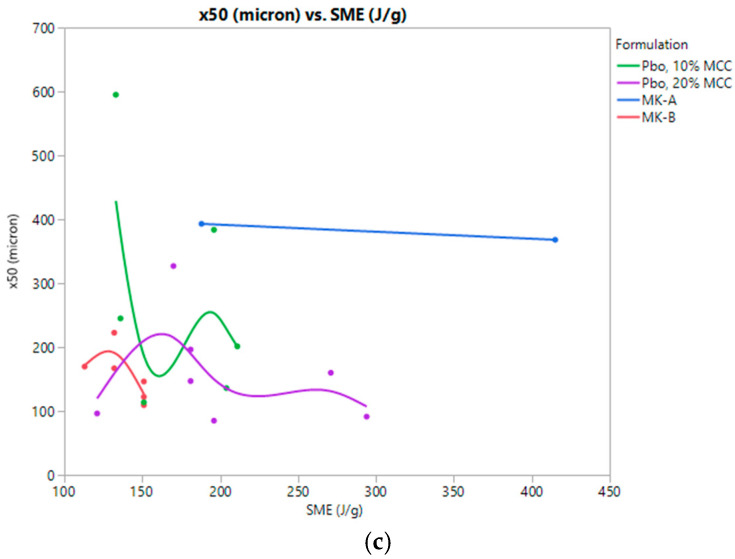
(**a**) Mean granule size by L/S; (**b**) Mean granule size by ST; (**c**) Mean granule size by SMESEM.

Table 7 lists key parameters for granule particle size distributions and the process parameters from the studied batches. Figure 3 shows the impact of these parameters on the formulations. Overall granule size trends were consistent with previous reports. Increasing L/S increased the extent of granulation and increased the mean of the granule size distribution overall. Increasing the specific throughput also increased the resulting granule size. With a higher fill level, the solids may be more compressed inside the barrel and experience more shear [23]. In this study, higher SME did not appear to impact the granule size as much as L/S or ST. Comparing the formulations, the MK-A formulation with a high lactose monohydrate level was readily granulated at low L/S, while placebo and MK-B formulations had similar granule sizes and impacts of TSWG process parameters.

**Figure 4:**
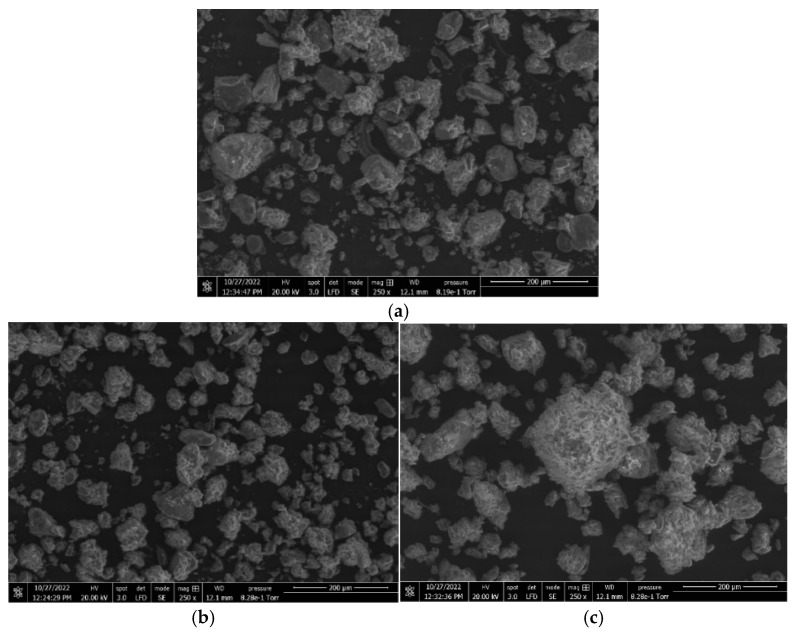

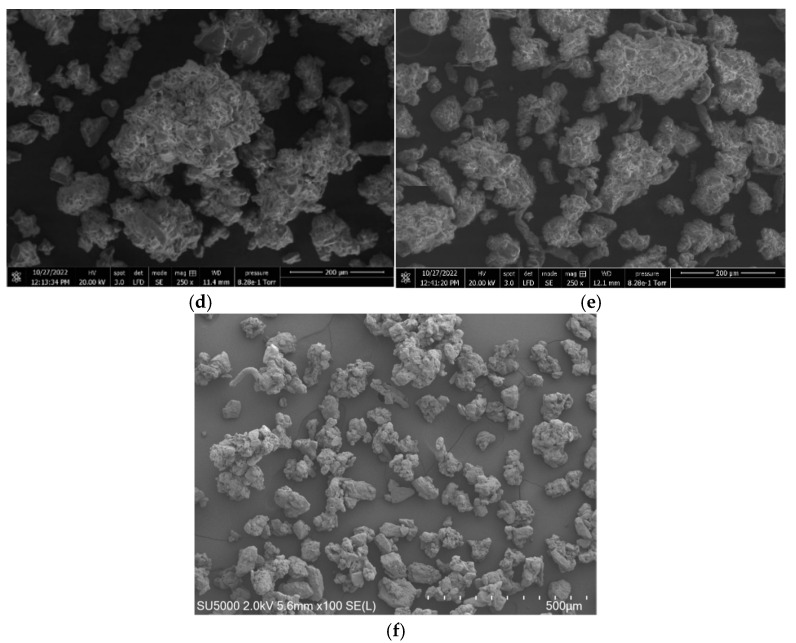
Scanning electron micrographs of (**a**) placebo granules, Run #37; (**b**) MK-B, Run #4; (**c**) MK-B, Run #6; (**d**) MK-A Run #1; (**e**) MK-A Run #2, all 250×. (**f**) MK-A FBG, 100×.

The scanning electron micrographs in Figure 4 show the size and morphology of the granules from the different formulations. The two micrographs of MK-B granules made with different process conditions show the difference between relatively low and high extents of granulation. The MK-A micrographs show the larger, rougher granules produced with that formulation, which contain a high amount of a water-soluble filler, lactose, without any MCC. The granule morphology is similar to granules produced using batch processes.

#### 3.2.2. Bulk and Tap Density

The granule bulk and tap densities for these runs are shown in Table 8. The TSWG granules are similar in density to the reference granules, depending on the process parameters. There is a weak trend for higher bulk and tap densities for granules with larger particle sizes. The Carr index values indicate that the granules are expected to have fair to passable flow.

#### 3.2.3. Tabletability

**Figure 5:**
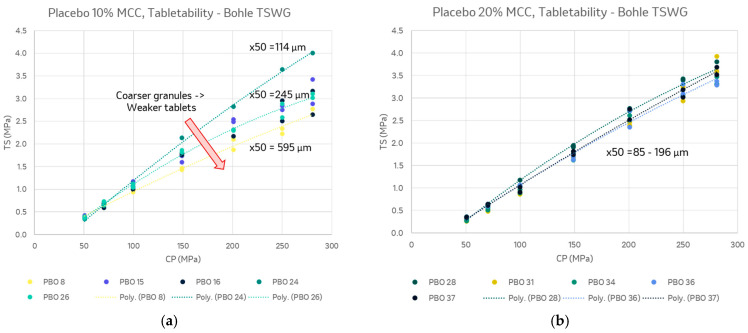
Tabletability of (**a**) 10% *w*/*w* MCC and (**b**) 20% *w*/*w* MCC placebo granules made with the QbCon1.

For the 10% *w*/*w* MCC placebo, the granule size varied more over the process conditions studied, and the tabletability trended with the mean granule size. The largest granules studied produced the weakest tablets, though still near the heuristic for high-quality tablets, with 2 MPa tensile strength at 200 MPa compaction pressure. The 20% *w*/*w* MCC placebo granule size was less sensitive to the liquid addition level. This may be related to the additional water uptake of MCC and replacement of 10% *w*/*w* lactose monohydrate, which readily granulates. The tabletability data are therefore more consistent across the samples and above target across the conditions studied (Figure 5).

**Figure 6:**
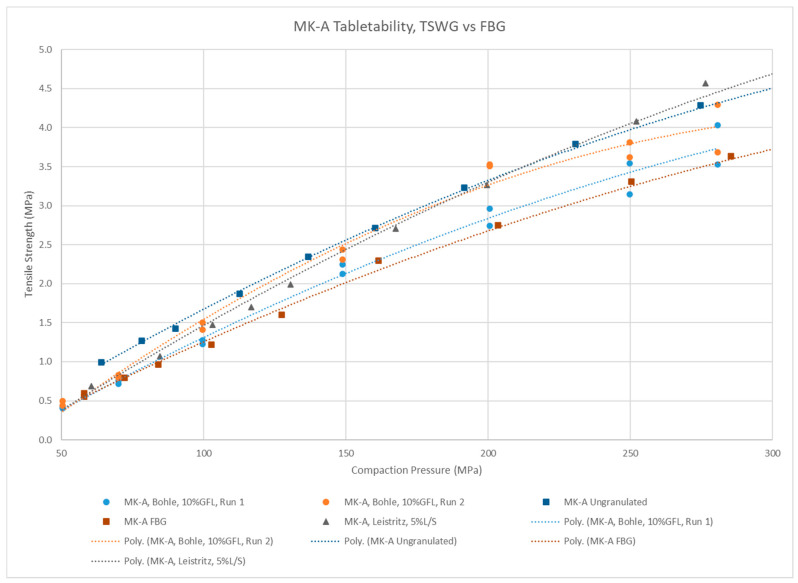
Comparing the tabletability of MK-A granulations made by continuous and batch wet granulation techniques.

MK-A granules made with the QbCon1 were compared with granules that were granulated with the Leistritz 18 mm and then tray dried, as well as fluid-bed granulated (FBG) granules of the same formulation (Figure 6). As previously mentioned, this formulation contains a significant amount of lactose, ~55% *w*/*w*, so it granulates at low liquid addition levels. The QbCon1 samples are similar in tableting performance to the Leistritz 18 mm and FBG samples, all of which are well above the target tablet strengths.

**Figure 7:**
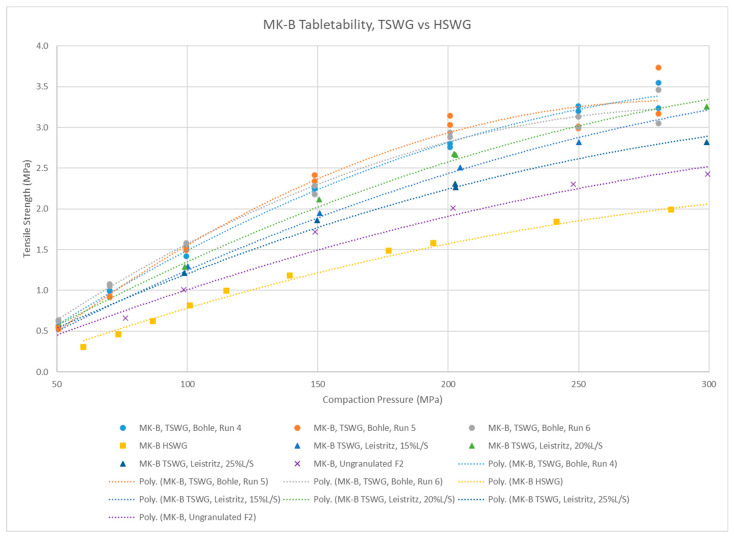
Comparing the tabletability of MK-B granulations made by continuous and batch wet granulation techniques.

The MK-B granules from the QbCon1 (F2, Table 4) were compared to previously made Leistritz 18 mm, tray-dried granules and high-shear wet granulated (HSWG), fluid-bed-dried granules (Figure 7). The reference MK-B HSWG granules were below target performance, with ~1.5 MPa tensile strength at 200 MPa compaction pressure. Note that the HSWG formulation differs (F1, Table 5) with extragranular MCC to aid compression. The QbCon1 granules are expected to be less dense, with more internal porosity [24]. This formulation shows some impact of TSWG parameters on tabletability, which may be linked to differences in granule internal porosity as well [22].

## 4. Conclusions

Placebo and active formulations were readily granulated and dried using QbCon1 equipment. The equipment design allows for the rapid adjustment of process conditions to generate high-quality granules that flow and compact well and to rapidly study the impact of different process conditions with only minimal required material consumption. The novel continuous dryer was able to dry granules at all conditions to <2% LOD within typical operating conditions and facilitated rapid process studies.

These granules were similar in performance with results from granules made by continuous granulation on the Leistritz 18 mm followed by tray drying and batch wet granulation and drying processes. Compared to batch techniques, the QbCon1 equipment allowed for much more rapid formulation and process studies. Granules made with the QbCon1 had similar tableting performance compared with FBG for one formulation and improved tableting performance versus HSWG for another. Differences in tabletability are expected to be related to differences in the mechanism of granulation, not the use of batch versus continuous granulation. Overall, this study demonstrates that conversion to continuous WG and drying was technically successful with the potential for improved tableting performance.

## Figures and Tables

**Table 1:** Placebo formulation comprised of lactose monohydrate, 10% or 20% *w*/*w* microcrystalline cellulose (MCC), and croscarmellose sodium.

Component	Level, % *w*/*w*
Microcrystalline cellulose	9.25–18.5
Lactose monohydrate	74–83.25
Croscarmellose sodium	3.0
Polyvinylpyrrolidone (PVP), Wet addition	4.0
Magnesium stearate, Extragranular	0.50

**Table 2:** MK-A formulation based on FBG reference.

Component	Level, % *w*/*w*
MK-A	33.3
Lactose monohydrate	55.7
Croscarmellose sodium, Intragranular	2.0
Polyvinylpyrrolidone (PVP), Wet addition	5.0
Croscarmellose sodium, Extragranular	3.0
Magnesium stearate, Extragranular	1.0

**Table 3:** MK-B formulation based on HSWG reference, Formulation 1 (F1).

Component	Level, % *w*/*w*
MK-B	20.0
Lactose monohydrate	40.0
Microcrystalline cellulose, Intragranular	5.1
Croscarmellose sodium, Intragranular	2.0
Polyvinylpyrrolidone (PVP), Wet addition	4.0
Microcrystalline cellulose, Extragranular	24.9
Croscarmellose sodium, Extragranular	2.0
Magnesium stearate, Extragranular	2.0

**Table 4:** MK-B formulation modified for TSWG, Formulation 2 (F2).

Component	Level, % *w*/*w*
MK-B	20.0
Lactose monohydrate	57.0
Microcrystalline cellulose	14.0
Croscarmellose sodium, Intragranular	3.0
Polyvinylpyrrolidone (PVP), Wet addition	4.0
Magnesium stearate, Extragranular	2.0

**Table 5:** Granulation and drying conditions and resulting LOD, Pbo = Placebo. Note one sample has >2% LOD (*).

Formulation	Solid Feed Rate (kg/h)	L/S (g Liquid/g Solid)	Screw Speed (rpm)	Volume of Dryer Air (STP m^3^/h)	Temperature of Dryer Air (°C)	Acceleration of Dryer (m/s^2^)	% LOD
Pbo, 10% MCC Run 8	2	30	80	27	90	4.7	2.15 *
Pbo, 10% MCC Run 12	3	30	120	35	90	3	1.8
Pbo, 10% MCC Run 15	2	20	80	17	90	4	0.83
Pbo, 10% MCC Run 16	2	20	120	15	90	5	1.96
Pbo, 10% MCC Run 24	2	10	80	10	80	4	1
Pbo, 10% MCC Run 26	2	10	120	10	80	3	1.65
Pbo, 20% MCC Run 27	2	10	80	10	80	4.5	1.15
Pbo, 20% MCC Run 28	2	10	120	10	90	4.5	1.97
Pbo, 20% MCC Run 31	2	20	80	18	90	4	1.52
Pbo, 20% MCC Run 34	2	20	120	17	90	4	1.6
Pbo, 20% MCC Run 36	2	30	80	28	90	4.3	1.86
Pbo, 20% MCC Run 37	2	30	120	28	90	4.3	1.48
Pbo, 20% MCC Run 38	3	30	90	35	90	4	1.93
MK-A Run 1	0.5	10	100	10	70	3.5	0.31
MK-A Run 2	2	10	100	15	70	4.5	0.36
MK-B F1 Run 1	1	17.8	50	11	60	5	1.51
MK-B F1 Run 2	1	14.2	50	10	50	5.5	1.22
MK-B F2 Run 3	1	10	35	10	40	7	1.54
MK-B F2 Run 4	1	25	40	13	70	4.5	1.57
MK-B F2 Run 5	1	20	40	11	60	4.5	1.77
MK-B F2 Run 6	1	20	40	10	60	6	1.08

**Table 6:** Mean residence times in continuous dryer.

Drying Air Acceleration (m/s^2^)	Mean Residence Time (s)
4	104
6	40.7
8	24.8

**Table 7:** Dried, milled granule particle size distribution, reported means of *n* = 3.

Sample	×10	×50	×90	L/S (g Liquid/g Solid)	ST (g/rev)	SME (J/g)
Pbo, 10% MCC Run 8	95	595	1845	30	0.417	133
Pbo, 10% MCC Run 12	85	384	2236	30	0.417	196
Pbo, 10% MCC Run 15	59	201	1961	20	0.417	211
Pbo, 10% MCC Run 16	60	136	1223	20	0.278	204
Pbo, 10% MCC Run 24	48	114	1064	10	0.417	151
Pbo, 10% MCC Run 26	63	245	1244	10	0.278	136
Pbo, 20% MCC Run 27	46	96	929	10	0.417	121
Pbo, 20% MCC Run 28	54	147	1038	10	0.278	181
Pbo, 20% MCC Run 31	41	85	738	20	0.417	196
Pbo, 20% MCC Run 34	49	91	745	20	0.278	294
Pbo, 20% MCC Run 36	56	196	1250	30	0.417	181
Pbo, 20% MCC Run 37	65	160	1032	30	0.278	271
Pbo, 20% MCC Run 38	64	327	1769	30	0.556	170
MK-A Run 1	63	368	1515	10	0.083	415
MK-A Run 2	85	393	1791	10	0.333	188
MK-B Run 1	65	223	1472	17.8	0.333	132
MK-B Run 2	58	170	1143	14.2	0.333	113
MK-B Run 3	53	167	1179	10	0.476	132
MK-B Run 4	53	146	1117	25	0.417	151
MK-B Run 5	48	122	1005	20	0.417	151
MK-B Run 6	47	109	1243	20	0.417	151

**Table 8:** Bulk and tap densities for QbCon1 runs and reference granules.

**Placebo**			
**Run**	**Bulk (g/mL)**	**Tap 500 (g/mL)**	**Carr Index**
HSWG/FBD	0.47	0.62	25
8	0.41	0.55	26
12	0.42	0.55	24
15	0.47	0.60	22
16	0.46	0.60	24
23	0.48	0.67	28
24	0.47	0.63	26
26	0.51	0.67	25
27	0.49	0.66	25
28	0.48	0.67	28
31	0.45	0.61	26
34	0.44	0.58	24
36	0.42	0.57	26
37	0.44	0.57	22
38	0.46	0.59	21
**MK-A**			
**Run**	**Bulk (g/mL)**	**Tap 500 (g/mL)**	**Carr Index**
FBG	0.54	0.68	21
TSWG, Leistritz 18 mm	0.59	0.73	19
1	0.48	0.65	26
2	0.50	0.63	20
**MK-B**			
**Run**	**Bulk (g/mL)**	**Tap 500 (g/mL)**	**Carr Index**
HSWG/FBD	0.58	0.72	20
1	0.49	0.63	23
2	0.51	0.63	20
3	0.53	0.69	23
4	0.49	0.62	21
5	0.48	0.64	24
6	0.49	0.63	22

## Data Availability

The data that support the findings of this study are available from the corresponding author, S.P.F., upon reasonable request.
